# A Dynamical Model of Equatorial Magnetosonic Waves in the Inner Magnetosphere: A Machine Learning Approach

**DOI:** 10.1029/2020JA028439

**Published:** 2021-06-21

**Authors:** R. J. Boynton, S. N. Walker, H. Aryan, Y. Hobara, M. A. Balikhin

**Affiliations:** ^1^ Department of Automatic Control and Systems Engineering University of Sheffield Sheffield UK; ^2^ University of California, Los Angeles Los Angeles CA USA; ^3^ Department of Computer and Network Engineering University of Electro‐Communications Tokyo Japan

**Keywords:** magnetosonic waves, machine learning, NARMAX

## Abstract

Equatorial magnetosonic waves (EMS), together with chorus and plasmaspheric hiss, play key roles in the dynamics of energetic electron fluxes in the magnetosphere. Numerical models, developed following a first principles approach, that are used to study the evolution of high energy electron fluxes are mainly based on quasilinear diffusion. The application of such numerical codes requires statistical models for the distribution of key magnetospheric wave modes to estimate the appropriate diffusion coefficients. These waves are generally statistically modeled as a function of spatial location and geomagnetic indices (e.g., AE, Kp, or Dst). This study presents a novel dynamic spatiotemporal model for EMS wave amplitude, developed using the Nonlinear AutoRegressive Moving Average eXogenous machine learning approach. The EMS wave amplitude, measured by the Van Allen Probes, are modeled using the time lags of the solar wind and geomagnetic indices as inputs as well as the location at which the measurement is made. The resulting model performance is assessed on a separate Van Allen Probes data set, where the prediction efficiency was found to be 34.0% and the correlation coefficient was 56.9%. With more training and validation data the performance metrics could potentially be improved, however, it is also possible that the EMS wave distribution is affected by stochastic factors and the performance metrics obtained for this model are close to the potential maximum.

## Key Points

- Machine learning is used to develop a novel dynamical model of the magnetosonic waves using solar wind, geomagnetic indices and location
- This model is important because it can potentially lead to an improvement to the forecasting capabilities of numerical radiation belt models
- The performance metrics of the model could potentially be improved with more data or the system could be affected by stochastic factors

## 1 Introduction

The dynamics and evolution of the radiation belt environment is becoming a more important area of interest to understand for our modern technological society as it becomes more reliant on space based infrastructure. Usage of efficient electric propulsion for the transfer of spacecraft to geostationary orbit has led to a significant increase in time that such spacecraft spend in the heart of the radiation belts, increasing their susceptibility to the adverse effects of energetic electrons. Reliable forecasts of the evolution of energetic electron fluxes in the magnetosphere can potentially assist in the mitigation of these adverse effects on spacecraft operation.

Models that are partially based on first principles employ numerical codes that involve finding solutions of the diffusion equations, such as the Comprehensive Inner Magnetosphere Ionosphere (CIMI) model Fok et al (2014) and the British Antarctic Survey Radiation Belt Model (BAS‐RBM) Horne, Glauert et al. (2013). These models require an estimate of the electron flux boundary conditions and the calculation of diffusion coefficients. There are a number of different approaches used to calculate the diffusion coefficients based on quasilinear theory, which require an estimate of the amplitude of various wave types Summers (2005); Albert (2008); Mourenas et al. (2012); Mourenas and Ripoll (2012). Most of these models are statistical distributions of the wave amplitudes, parameterized by the location of observations and current values for geomagnetic indices Meredith et al. (2001); Glauert and Horne (2005); Pokhotelov et al. (2008); Li et al. (2011); Agapitov et al. (2011); Meredith et al. (2012); Horne, Kersten et al. (2013); Mourenas et al. (2013); Gao et al. (2014); Mourenas et al. (2016). Such a parameterization has an underlying assumption that only the instantaneous activity of geomagnetic indices and solar wind values influence the wave distribution and the preceding state of the magnetosphere has no role. However, many studies have shown that the electron fluxes at Geostationary Earth Orbit (GEO) are influenced more by changes in the solar wind than geomagnetic indices Paulikas and Blake (1979); Blake et al. (1997); Reeves et al. (2011); Balikhin et al. (2011); Boynton et al. (2013, 2015); Boynton, Balikhin et al. (2016) and also that these parameters are temporally lagged with respect to evolution of the electron fluxes Li et al. (2005); Balikhin et al. (2012); Boynton et al. (2013); Boynton, Balikhin et al. (2016). Thus, such parameters that are statistically related to the fluences of electrons should also be included in the development of wave models. This motivated the development of the wave models by Aryan et al. (2014), in which the waves were parameterized according to time delayed observations of the solar wind, and were subsequently extended to multi‐parameter chorus and hiss wave models Aryan et al. (2016, 2017). Boynton et al. (2018) investigated the most significant solar wind and geomagnetic index control parameters for lower band chorus (LBC) waves using the error reduction ratio (ERR) technique. The ERR is able to assess a wide range of nonlinearities from the solar wind and geomagnetic indices and their respective time lags. Boynton et al. (2018) found that the AE index coupled with the solar wind velocity controlled the evolution of the LBC waves throughout most of the inner magnetosphere, especially in the regions where LBC waves are generally observed.

In this study, equatorial magnetosonic (EMS) waves measured by the Van Allen Probes spacecraft are modeled using Nonlinear AutoRegressive Moving Average eXogenous (NARMAX) machine learning techniques.

The EMS waves are whistler mode emissions that propagate almost perpendicular with respect to the external magnetic field and are observed both inside and outside the plasmasphere Russell et al. (1970); Laakso et al. (1990). It is widely accepted that EMS waves are predominantly confined to approximately 3° of the magnetic equator Russell et al. (1970); Laakso et al. (1990); Cornilleau‐Wehrlin et al. (2003); Nemec et al. (2005), though, it has been shown that some EMS waves may be observed at higher latitudes Aryan et al. (2019). They are observed between the proton gyrofrequency and the lower hybrid resonance frequency and generated as a result of proton ring distributions formed during magnetic storms at ring current energies of the order of 10 keV Perraut et al. (1982); Boardsen et al. (1992); Chen et al. (2011); Ma et al. (2014); Balikhin et al. (2015). It has been shown that EMS waves are able to interact with electrons through Landau resonance and accelerate electrons to relativistic speeds Horne et al. (2007). Horne et al. (2007) found that the bounce and drift averaged energy diffusion rates for magnetosonic waves are comparable to those for whistler mode chorus. Boardsen et al. (2016) performed a statistical survey of the fast magnetosonic wave mode detected by the Van Allen Probes mission and found that the overall intensity of EMS waves increases with AE index. Mourenas et al. (2013) presented simplified analytical expressions of the pitch angle and momentum quasi‐linear diffusion rates of magnetospheric electrons in the presence of fast magnetosonic waves and demonstrated a good precision over a wide energy range between 100 keV and 2 MeV.

The motivation of this paper is to develop the first dynamic spatiotemporal model of the EMS waves. This could potentially be employed in numerical codes that involve finding solutions of the diffusion equations, replacing the statistical wave models with dynamic wave models. More accurate dynamical wave models should increase the accuracy of numerical codes that predict the radiation belt electron fluxes.

## 2 Data

The wave data were collected by the Van Allen Probes, which had a highly elliptical orbit with perigee ∼1.1 *R*_*E*_, apogee ∼5.9 *R*_*E*_, an inclination of 10.2°, and period of ∼9 h Mauk et al. (2014). The period considered in this study spanned from January 01, 2013 to December 31, 2017, where the model was trained on data from January 01, 2013 to December 31, 2015 and validated on data from January 01, 2016 to December 31, 2017. The background magnetic field measurements come from the fluxgate magnetometer (FGM), sampling at 64 Hz while the wave data comes from survey data from the WFR instrument, generating a full spectral matrix for 65 quasi‐logarithmically spaced frequency channels in the range ∼2 Hz–∼11 kHz every 6 s. These field instruments are part of the Van Allen Probes Electric and Magnetic Field Instrument Suite and Integrated Science (EMFISIS) Kletzing et al. (2013).

EMS waves are observed close to harmonics of the proton gyrofrequency (*ω*_*p*_) up to the lower hybrid frequency (*ω*_LH_). They exhibit almost linear polarization (ellipticity *ϵ* < 0.2), propagate perpendicular to the local magnetic field, typically with 88° < *θ*_Bk_ < 90°, and a high magnetic compressibility (*δB*_‖_/*B*_0_) since the wave magnetic field is directed is oriented along the local magnetic field. Occurrences of EMS Waves were identified using a similar method to Boardsen et al. (2016). The search criteria used were that the emissions occurred in the frequency range *ω*_*p*_ < *ω* < *ω*_*LH*_ and that the compressibility δ*B*_‖_/*B*_0_ > 0.7. This latter criterion proved better at identifying EMS waves than the application of criteria based on the ellipticity or propagation direction. The search criteria were used to generate mask arrays indicating the occurrence or absence of waves. A blob analysis was then employed to determine the frequency/time limits of the waves. Blob analysis is a technique borrowed from the field of computer vision, which analyses a binary image (an image containing two states/colors) to determine the number and size of continuous areas of one of the image values. A blob analysis was used to determine the areas in the binary spectrogram where the search criteria were met. A further constraint, namely the minimum area for each blob was set to remove the “noise” due to single pixels or small areas on the binary image that fulfill the search criteria. Finally, a nearest neighbors analysis was performed to ensure that the areas detected by the blob analysis extended in both time and frequency space, which removes any single bad spectra. The output is a list of times of individual spectra and the frequency range in which EMW were observed. For each spectra identified as containing EMS waves the maximum amplitude and its occurrence frequency were recorded. For spectra with no EMS waves, fill values of 10^−2^ pT were recorded, the reason for this being that it is important to know when EMS waves do and do not occur when training the model. This spectral information was then combined with satellite ephemeris data where values for the McIlwain *L*‐shell and Roederer *L* were based on the satellite location and field line mapping using the Olsen‐Pfitzer quiet time model Olson and Pfitzer (1982). The Olsen and Pfitzer model was used for as it provides a good model for the average external magnetic field value in comparison to measurements Friedel et al. (2005). This model was also adopted by the Panel for Radiation Belt Environment Modeling for improving space radiation models at the time when the data was developed and has been used in in a number of studies Meredith et al. (2012, 2018); Meredith, Horne, Kerstal et al. 2014; Meredith, Horne, Li et al. 2014. The wave amplitudes, together with spatial locations in *L*‐shell, magnetic local time (MLT), and Magnetic LATitude (MLAT), were then resampled with a resolution of 1 h.

Both solar wind and geomagnetic index data used in this study were taken from 1‐minute OMNIweb solar wind data (https://omniweb.gsfc.nasa.gov/ow_min.html). Where the solar wind data were from the Advanced Composition Explorer and WIND spacecraft, which are propagated to the bow shock. This was then averaged into a 1 h resolution. The 1 h time resolution was mainly chosen due to the error in propagation from L1 to the Earth, which increases as the time resolution increases.

## 3 Methodology

In this study, NARMAX methodology is employed to model the EMS waves. The NARMAX model was initially proposed by Leontaritis and Billings (1985a, 1985b) and is defined by

(1) $$\begin{array}{ccc} \hat{y}\left(t\right) & = & F\left[y\left(t-1\right),\;\ldots ,y\left({t-n}_{y}\right),\right.\\ & & \left.u_{1}\left(t-1\right),\;\ldots ,u_{1}\left({t-n}_{u_{1}}\right),\;\ldots ,\right.\\ & & \left.u_{m}\left(t-1\right),\;\ldots ,u_{m}\left({t-n}_{u_{m}}\right),\;\ldots ,\right.\\ & & \left.e\left(t-1\right),\;\ldots ,e\left({t-n}_{e}\right)\right]+e\left(t\right) \end{array}$$

where an estimate of the output $\hat{y}$ at time *t* is a nonlinear function *F* of past outputs *y*, inputs *u*, and residuals *e* ($e\left(t\right)=y\left(t\right)-\hat{y}\left(t\right)$); *m* is the number of inputs; and *n* are the respective lags.

The nonlinear function *F* can be represented by polynomials, rationals, wavelets, among others Billings (2013). In this study, the nonlinear function is set to be a polynomial. When *F* is expanded to a high degree polynomial there will be many monomials, most of which are very similar to each other and many which may not influence the system. In a polynomial model, these monomials will consist of linear and nonlinear coupled inputs, outputs, and noise terms of different lags up to the degree of polynomial selected for the model (e.g., *u*_2_(*t* − 1), *u*_1_(*t* − 1)*u*_3_(*t* − 1)*u*_4_(*t* − 2), *u*_1_(*t* − 1)*u*_3_(*t* − 4)*e*(*t* − 2) *y*(*t* − 1)*e*(*t* − 2), etc.). The Forward Regression Orthogonal Least Squares (FROLS) Billings et al. (1989) is used to find a small subset of monomials from the polynomial (often referred to as the term dictionary) that best represent the system. The FROLS algorithm optimizes the driving parameters and their coefficients by searching for the most influential the monomials in the expanded function F by using the ERR. In the next step, all other monomials are orthogonalized relative to the selected monomial and and the orthogonalized monomial with the highest ERR is selected. This process of selecting the monomial, which is orthogonalized to all the previously selected monomials, with the highest ERR is repeated until a criteria is satisfied. In this study, the Adjustable Prediction Error Sum of Squares criteria is employed Billings and Wei (2008). The NARMAX procedure then involves the statistical validation of the model using correlation tests Billings and Voon (1986); Billings and Zhu (1989, 1995). These correlations check that the residuals of the model are unrelated to the inputs. If there is a correlation between the residuals and the inputs, the tests will indicate if there is either a biased term within the model that needs to be removed or identify any nonlinear inputs missing from the model.

The NARMAX FROLS methodology was initially developed for control systems engineering problems but has since been employed in a diverse range of scientific fields. For example, NARMAX has been applied to analyzing the adaptive changes in the photoreceptors of Drosophila flies Friederich et al. (2009) and has been used to model the tide in the Venice Lagoon Wei and Billings (2006). In space physics, NARMAX models have been developed for the Dst index Boaghe et al. (2001); Balikhin et al. (2001); Boynton, Balikhin, Billings, Sharma et al. (2011) and the electron fluxes at GEO Wei et al. (2011); Boynton et al. (2015, 2019); Boynton, Balikhin et al. (2016). Boaghe et al. (2001) and Balikhin et al. (2001) transformed the NARMAX models to the frequency domain to examine the spectral properties of the Dst index dynamics. Since NARMAX models are physically interpretable, unlike most other machine learning techniques, it has also been used to develop a solar wind‐magnetosphere coupling function for the Dst index Balikhin et al. (2010); Boynton, Balikhin, Billings, Sharnma et al. (2011) and automatically determine the most influential inputs to a wide range of electron flux energies at GEO Balikhin et al. (2011, 2012); Boynton et al. (2013); Boynton, Mourenas et al. (2016 )and *L* = 4.2 *R*_*E*_ Boynton et al. (2017) and LBC waves Boynton et al. (2018).

The solar wind inputs were the velocity *v*, density *n*, dynamic pressure *p*, and the interplanetary magnetic field (IMF) factor deduced by Boynton, Balikhin, Billings, Wei et al. (2011) *B*_*f*_ = *B*_*T*_  sin^6^(θ/2) (where $B_{T}=\sqrt{B_{y}^{2}+B_{z}^{2}}$ and *θ* = arctan *B*_*y*_/*B*_*z*_). These solar wind and geomagnetic indices were chosen as inputs due to past statistical wave models. These wave models are often parameterized by AE index Meredith et al. (2001) and also by solar wind values Aryan et al. (2016). Aryan et al. (2016) also showed an asymmetry in the *B*_*z*_ component between north and south IMF, which is why *B*_*f*_ is chosen as an input as this variable takes into account the north‐south asymmetry due to dayside reconnection. One of the advantages of the NARMAX procedure is multiple inputs can used to define the initial model. If any of these inputs has no relation to the output, this input will not be selected in the final model. Since the EMS waves are generated as a result of proton ring distributions formed during magnetic storms at ring current energies Balikhin et al. (2015); Perraut et al. (1982), the SYM‐H index, a ring current index and a metric for magnetic storms, was chosen as an input to the model.

The lags employed for the solar wind inputs were 0, 1, 2, …, 12, 14, 16, …, 24, 28, 32, …, 48, so, put another way the lags were every hour between 0 to 12 h, every 2 h between 12 to 24 h, and every 4 h between 24 to 48 h. These solar wind parameters were time shifted to the bowshock and thus zero time lag can be employed as inputs. The geomagnetic indices used were SYM‐H and the AE index and the time lags employed were 1, 2, 3, …, 12, 14, 16, …, 24, 28, 32, …, 48, similar to the solar wind inputs with the exclusion of the zero time lag. The zero time lag was not included for the geomagnetic indices as the model is aiming to be a forecast model. A wide range of lags were chosen for the initial NARMAX model. Again, if any of these time lags do not play a role in the evolution of the EMS waves then the FROLS algorithm will not select these lags in the final model and the correlation tests in model validation will not indicate the missing inputs with these lags.

EMS are complicated to model from data as the distribution of the waves evolve in space as well as time. One solution to this problem is to include the spatial position of the measurement as an input to the model. Therefore, the instantaneous position of the spacecraft at the time of measurement was used as an input. This includes *L*‐shell *L*; sine and cosine of the MLT; and cosine of the MLAT.

In this study, the nonlinear function *F* was set to be a third degree polynomial. Initially, a second degree polynomial was trialed, however, during the statistical validation stage of the NARMAX model training, it was evident that there were many missing nonlinearities in the model as the correlation tests for most inputs with the residuals were not satisfied. The correlation tests were satisfied when the polynomial degree was increased to three, after a number of other small adjustments to the model.

For example, the cosine of the MLAT combined to the cubic power, however, during the statistical validation stage of the NARMAX model training, the correlation tests indicated a missing nonlinear cos(MLAT) at zero time lag, even though the model had selected a cube of the cos(MLAT) in the model. Therefore, the cube of cos^3^(MLAT) was employed in the model for the next iteration of the FROLS algorithm instead of cos(MLAT) to check if higher powers of the cos(MLAT) was missing from the model. This would allow the model to select up to the ninth power of cos(MLAT). In a subsequent run cos^3^(MLAT) then combined in the NARMAX models to higher powers indicating the correlation tests were correct in identifying this missing higher power of the cos(MLAT) input. However, the correlation tests still indicated a missing cos(MLAT) at zero time lag. Eventually cos^6^(MLAT) was settled on for the input to the model instead of cos(MLAT) as this satisfied the correlation tests. This is most likely due to EMS waves occurring in a narrow band around the geomagnetic equator where intensity decreases sharply the further away from the equator Russell et al. (1970); Laakso et al. (1990); Cornilleau‐Wehrlin et al. (2003); Nemec et al. (2005); Aryan et al. (2019).

Therefore, the NARMAX model used in this study for the EMS wave amplitude *B*_*w*_ was of the form

(2) $$\begin{array}{ccc} B_{w}\left(t\right) & = & F\left[v\left(t\right),v\left(t-1\right),\;\ldots ,v\left(t-48\right),\right.\\ & & \left.n\left(t\right),n\left(t-1\right),\;\ldots ,n\left(t-48\right),\;\ldots ,\right.\\ & & \left.p\left(t\right),p\left(t-1\right),\;\ldots ,p\left(t-48\right),\;\ldots ,\right.\\ & & \left.B_{f}\left(t\right),B_{f}\left(t-1\right),\;\ldots ,B_{f}\left(t-48\right),\;\ldots ,\right.\\ & & \left.SYM-H\left(t-1\right),SYM-H\left(t-2\right),\;\ldots ,SYM-H\left(t-48\right),\;\ldots ,\right.\\ & & \left.AE\left(t-1\right),AE\left(t-2\right),\;\ldots ,AE\left(t-48\right),\;\ldots ,\right.\\ & & \left.L\left(t\right),\cos\left(MLT\left(t\right)\right),\sin\left(MLT\left(t\right)\right),\cos^{6}\left(MLAT\left(t\right)\right)\right] \end{array}$$

It should be noted that the autoregressive terms and moving average terms are excluded from this model as the spacecraft does not return to exactly the same position on each orbit. In this case, the model reduces to a volterra series model (only consists of the nonlinear exogenous input terms).

## 4 Model and Performance

The EMS wave model was trained on Van Allen Probe‐A data from January 01, 2013 to December 31, 2015 and the resultant model was then validated on separate Van Allen Probe‐A data from January 1, 2016 to December 31, 2017, which is just over one apsidal period of the Van Allen Probes giving a full MLT coverage. Here, solar wind and geomagnetic indices from the period were used as inputs to the model, along with the spatial co‐ordinates of Probe‐A to compute an estimate of the EMS waves along the track of Probe‐A.

The performance of the model was evaluated using two metrics; the correlation coefficient (CC) and the prediction efficiency (PE) and performed on the logarithm of the EMS wave amplitude. The CC is defined by Equation 3 and the PE by Equation 4. These performance metrics have been applied in many previous studies for assessing geospace models Baker et al. (1990); Klimas et al. (1996); Temerin and Li (2006); Wei et al. (2004); Zhu et al. (2007); Amariutei and Ganushkina (2012).

(3) $$\rho =\frac{\sum_{t=1}^{N}\left[\left(y\left(t\right)-\bar{y}\right)\left(\hat{y}\left(t\right)-\bar{\hat{y}}\right)\right]}{\sqrt{\sum_{t=1}^{N}\left[{\left(y\left(t\right)-\bar{y}\right)}^{2}\right]\sum_{t=1}^{N}\left[{\left(\hat{y}\left(t\right)-\bar{\hat{y}}\right)}^{2}\right]}}100\%$$

(4) $$E_{PE}=\left[1-\frac{\sum_{t=1}^{N}\left[{\left(y\left(t\right)-\hat{y}\left(t\right)\right)}^{2}\right]}{\sum_{t=1}^{N}\left[{\left(y\left(t\right)-\bar{y}\right)}^{2}\right]}\right]100\%$$

The CC for the validation period was 56.9% and the PE was 34.0%.

**Figure 1:**
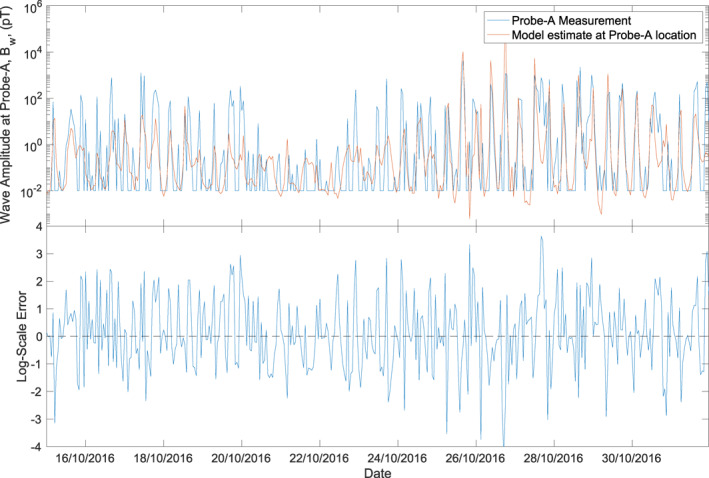
Top panel shows the equatorial magnetosonic wave amplitude measured by the Van Allen Probes‐A as it tracks through it's orbit (blue) and the estimate by the model at the location of Probe‐A (orange) from October 15, 2016 to October 31, 2016. The error between the measured and model estimate is shown in the lower panel.

Figure 1 shows the comparison between the measured EMS (blue) wave amplitude and the estimate by the model (orange) for a short period from October 15 to 31, 2016 and the error between the two in the lower panel. The figure shows that the EMS wave amplitude exhibits peaks and troughs in the measured and estimated data that correspond to the periodic transit of the spacecraft in its orbit as it tracks from perigee to apogee, where the peaks in wave amplitude correspond to the perigee and troughs in wave amplitude correspond to a high *L* shell. In this 15 day period, there is an interval between October 21 and 22 during which the measured wave amplitude does not exceed 10 pT followed by an interval on October 25 in which the amplitudes increase to 10^4^ pT. These amplitude changes are successfully reproduced by the model. In this period, the lower cut off, when there are no EMS waves measured, is shown by the flat periods at 10^−2^ pT. If the model output includes the same on/off cut off of the EMS waves as the measured data, where model predicted EMS wave amplitude below 10^−2^ pT is set to 10^−2^ pT, the performance of the model improves slightly to a PE of 34.7% and a CC of 57.5%.

**Figure 2:**
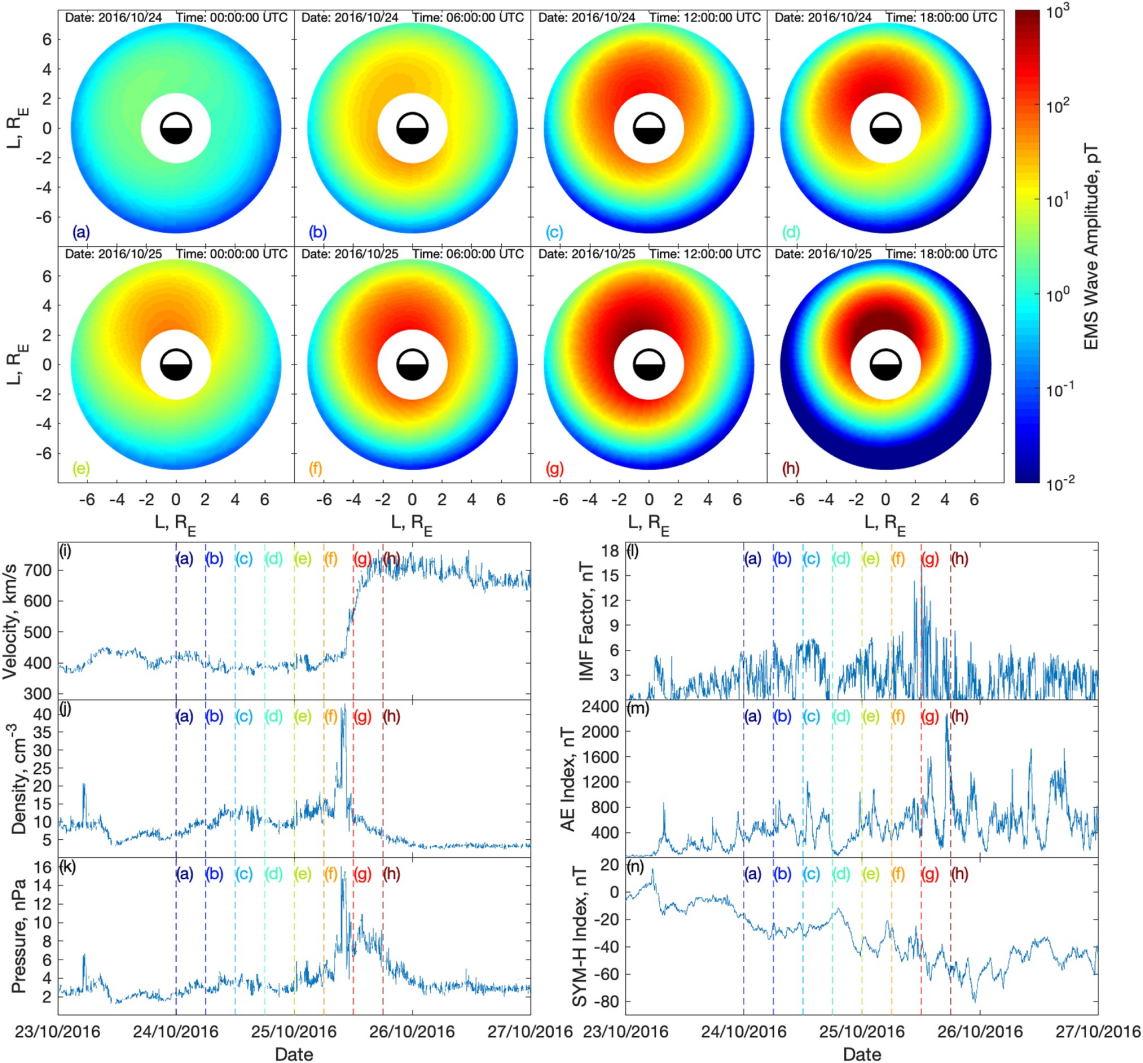
Snapshots of the model predicted equatorial magnetosonic (EMS) wave amplitude throughout the inner magnetosphere between 2 and 7 *R*_*E*_ at an MLAT of 0° are shown in panels (a–h) dating from 0000 UTC on October 24, 2016 to 1800 UTC on October 25, 2016. The inputs to the model are display in the remaining panels; solar wind velocity in panel (i), density in panel (j), dynamic pressure in panel (k), IMF factor in panel (l), AE index in panel (m) and SYM‐H index in panel (n). The vertical lines in panels (i–m) signifies the times of the eight EMS waves snapshots shown in panels (a–h). IMF, interplanetary magnetic field.

From the NARMAX EMS wave model, the wave amplitude can be mapped out to the whole inner magnetosphere by using all locations as spatial inputs for each time. From this we will be able to see how the EMS wave intensities evolve in space and time. Figure 2 shows snapshots of this reconstruction from 2 to 7 *R*_*E*_ at an MLAT of 0°, which is also shown as a video supplied in the supporting material. Figure 2 shows the EMS wave amplitude throughout the inner magnetosphere for 8 snapshots in panels (a–h) dating from 0000 UTC on October 24, 2016 to 1800 UTC on October 25, 2016. Panels (i–n) show the solar wind velocity, density, dynamic pressure, IMF factor, AE, and SYM‐H input parameters respectively. The vertical lines in panels (i–m) signifies the times of the eight EMS waves snapshots shown in panels (a–h). This figure illustrates how the the wave amplitude varies in time and space with changing solar wind and geomagnetic indices. In panel (a), there is low wave amplitude throughout the inner magnetosphere. The wave amplitude then builds through panels (a–d), from 0000 UTC to 1800 UTC October 24, 2016, where it can be seen that the AE index increases to remain over 400 nT 2 h prior to (a), the SYM‐H index starts to drop below −20 nT, IMF factor spikes are over 6 nT, solar wind density and dynamic pressure increase slightly after (a), while the solar wind velocity remain approximately constant at around 400 km/s. Prior to (d), there is a decrease in IMF factor and AE index, and an increase in SYM‐H, which leads to a reduction in EMS wave amplitude shown in panel (e). After (e), EMS wave amplitude build up again to panel (h) at 1800 UTC on October 25, 2016, during which IMF factor and AE index increase, SYM‐H decreases, while the other solar wind variables remain constant up until after (f), at which density, pressure and velocity all increase. From panel (g–h) the EMS wave amplitudes increase for *L* < 4 *R*_*E*_ on the dayside, while decreasing for *L* > 4 *R*_*E*_. This coincides with a decrease in AE index 2–3 h prior.

## 5 Discussion

The aim of this study was to investigate how machine learning can be applied to develop a dynamical model of EMS waves in the inner magnetosphere. The model has been used to map the evolution of the waves in time to help picture how they respond to changes in solar wind and geomagnetic indices, which is difficult to picture from tracks from satellite data or static statistical wave models.

As stated previously, the final EMS wave model was trained on Van Allen Probe‐A data from January 01, 2013 to December 31, 2015. Initially, a smaller training set was trialed for 2013 to the end 2014, however, this resulted in the MLT terms not being selected by the FROLS algorithm. This was probably due to the lack of MLT coverage from just 2 years of the Van Allen Probes mission, so that the algorithm did not have enough data to detect any relationship between EMS waves and MLT, since the apsidal period of the Van Allen Probes is just under 2 years. When the data set was expanded to the end of 2015, MLT was selected by the FROLS algorithm. Better results could be obtained if the model could be trained on two apsidal periods, however, this would leave less data for validation.

Using 3 years of data for training still means that there is only a small data set for validating the model from Van Allen Probe‐A data. Probe‐B could also be used for validation for the entirety of the mission period, since it is in a different location than Probe‐A, on which the model was trained. However, the Probe‐B data set can only be used if it differs from the Probe‐A data set. Probe‐B roughly follows the same path of Probe‐A, with Probe‐B in a slightly slower orbit in which the rate changes over time. This could mean the measurements from the two spacecraft may not differ by much on the hourly sampled time scales when the two spacecraft are close to each other but may differ significantly when the two spacecraft are far away from each over.

**Figure 3:**
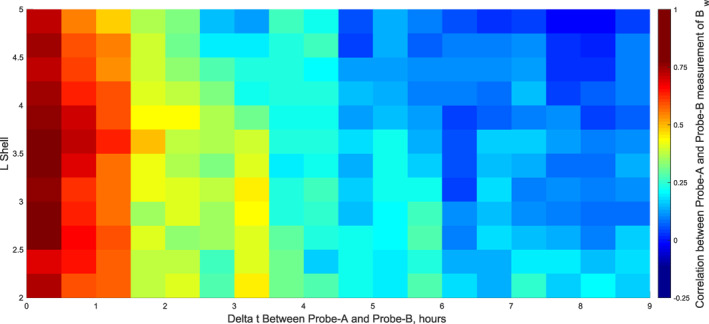
Autocorrelation of the equatorial magnetosonic (EMS) waves from Van Allen Probe‐A and Probe‐B measurements, with the time delay between the measurements of A and B on the *x* axis, the *L* shell on the *y* axis, and the correlation between the A and B measurements of EMS waves.

To see if the readings of A and B differ, depending on the time gap between the two spacecraft, the measurements of A were binned as the spacecraft passed intervals of the *L* shells for outbound and inbound tracks of the orbit. These intervals were from *L* = 2, 2.25, 2.5, …, 4.5, 4.75, 5. The measurement of the next pass of B at this *L* shell was then recorded and the time gap between A and B was noted. The wave amplitude measurements of A and B were then correlated as a function of *L* shell and time gap to see the autocorrelation of the EMS waves in space and time delay. Figure 3 shows the surface plot of the correlations between the A and B measurements of EMS waves, with the time delay between the measurements of A and B on the *x* axis, the *L* shell on the *y* axis. The figure shows a drop off in correlation after 1.5 h delay, which means that the measurements from A and B differ significantly enough to use both during periods where the spacecraft are separated by at least 1.5 h. Therefore, in future studies, datasets from A and B could be stitched together for training and validation of the model, potentially leading to models with increased performance.

It is also possible that even with more training data covering a wider spatial range the model performance may not increase. This would mean that the EMS wave distribution throughout the inner magnetosphere is not deterministic but are also largely impacted by stochastic factors.

The system may also have some highly nonlinear features that the polynomial NARMAX model struggles to capture. In this case, a different model structure could be trialed to see if there is a significant performance increase. One structure to try would be a rational NARMAX model Zhu and Billings (1993), which is defined the ratio of two polynomial NARMAX models. Another option is a wavelet‐NARMAX model Billings and Wei (2005), where a polynomial NARMAX model is used to characterize any smooth varying trends and a wavelet model is used to characterize any rapid dynamics.

The EMS wave model developed in this study could be integrated with numerical models of the radiation belts, such as CIMI Fok et al. (2014), BAS‐RBM Horne, Glauert et al. (2013), and others. Instead of calculating diffusion coefficients from the static statistical wave models, the diffusion coefficients could be calculated from the dynamical NARMAX EMS wave model. One issue is that many numerical codes run in adiabatic invariant space. As such, since the current model is in real space, a co‐ordinate transform from real to adiabatic co‐ordinates will be required for some models.

One of the main advantages of NARMAX methodologies over other machine learning techniques is that the models are interpretable, it is possible to inspect the terms or, in this case, the monomials that make up the polynomial NARMAX model. The monomials are selected one at a time by the FROLS algorithm using the ERR, where, at each step, the monomial with the highest contribution to the output variance (ERR) is selected as the model term. When inspecting the EMS wave model, the top terms in the model will have more contribution to the evolution of the waves than those at the bottom end of the model. The top terms here are the spatial locations, with *L* and cos^6^(MLAT) coupled with AE index making up the first and second terms of the model. This is understandable, as it can be seen in Figure 1 how the wave amplitude changes as the Van Allen Probes tracks through the orbit with changing *L*. Also, since EMS waves are mainly observed within ∼20° magnetic equator Aryan et al. (2019), MLAT should be an important factor, and that it is coupled to AE index could mean that larger disturbances in AE may lead to EMS waves occurring further away from the equator. The most influential non spatial input parameter is the AE index, which was also reported to be the main control parameter of the lower band chorus waves by Boynton et al. (2019).

## 6 Conclusions

The NARMAX machine learning technique has been applied to develop a dynamical spatiotemporal model of the EMS waves in the inner magnetosphere. The model was then used to map out the dynamical EMS wave response across the inner magnetosphere to changing solar wind conditions.

The performance of the model has been tested on Van Allen Probes data, which resulted in a PE of 34.0% and CC of 56.9%. Compared to other signals in space weather, such as modelling the Dst index or electron fluxes Wei et al. (2004); Boynton, Balikhin, Billings, Sharma et al. (2011); Boynton et al. (2015), the EMS wave model performance metrics are not as high. With more training and validation data, which cover a wider spatial range, the performance of a data based machine learning model could potentially be improved. This could involve stitching together various datasets from various past and present missions. However, it is also possible that the EMS wave distribution is more affected by stochastic factors than other signals and thus the performance metrics obtained by the model deduced in this study are close to the potential maximum.

## Supporting information

Supporting Information S1

Movie S1

## Data Availability

The Van Allen Probes EMFISIS data used in this study were obtained from https://emfisis.physics.uiowa.edu/data/index. The solar wind and geomagnetic index data were from OMNIweb (https://omniweb.gsfc.nasa.gov/ow_min.html). The output of the model developed during this study will be made available at http://www.ssg.group.shef.ac.uk/USSW/UOSSW.html.
