# Supplementary material for: A Dynamical Model of Equatorial Magnetosonic Waves in the Inner Magnetosphere: A Machine Learning Approach
Source: J Geophys Res Space Phys. 2021 Jun 21;126(6):e2020JA028439. doi: 10.1029/2020JA028439 (PMC11578165; doi:10.1029/2020JA028439)
Supplement: Supplementary file 1 — Supporting Information S1 [file JGRA-126-0-s001.pdf]

Supporting Information for “A dynamical model of equatorial magnetosonic waves in the inner magnetosphere: A machine learning approach”

R. J. Boynton,[1] S. N. Walker,[1] H. Aryan,[1,2] Y. Hobara,[3] M. A. Balikhin[1]

Additional Supporting Information (Files uploaded separately)

1. Captions for Movie MS1 (ms01.mp4).

Introduction

[1]Department of Automatic Control and Systems Engineering, University of Sheffield, Sheffield S1 3JD, United Kingdom.

[2]University of California Los Angeles, Atmospheric and Oceanic Sciences, Math Sciences Building, Los Angeles, CA 90095-1565, United States.

[4]Department of Computer and Network Engineering, The University of Electro-Communications, Tokyo, Japan.

Movie MS1.

Movie showing the Model estimated Equatorial Magnetosonic waves from 00:00 UT 1 October 2016 to 00:00 UT 1 November 2016 with solar wind and geomagnetic index inputs; velocity, density, pressure, IMF factor, SYM-H index and AE index.
